# Supplementary figures and images for: Clinical and pathological characterization of Central Nervous System cryptococcosis in an experimental mouse model of stereotaxic intracerebral infection
Source: PLoS Negl Trop Dis. 2023 Jan 19;17(1):e0011068. doi: 10.1371/journal.pntd.0011068 (PMC9888703; doi:10.1371/journal.pntd.0011068)

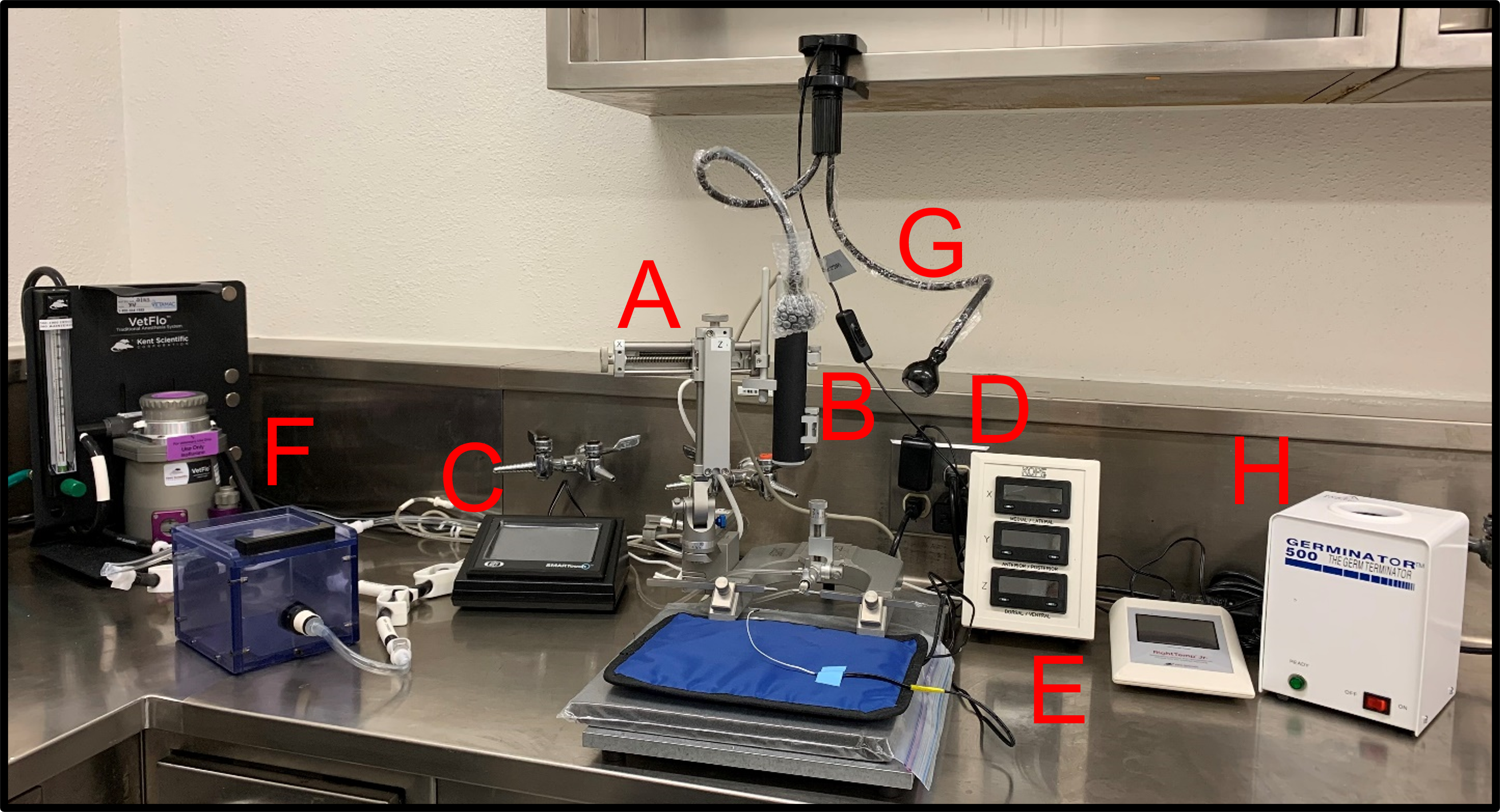

Supplement: S1 Fig — (A) Stereotaxic instrument uses a three-dimensional coordinate system (e.g., The Allen Mouse Brain Atlas; https://mouse.brain-map.org/static/atlas) to locate specific targets inside the mouse brain. (B) Ultra micro pump holds microinjection syringes to deliver picoliter to milliliter volumes and mounts directly on the stereotaxic frame. (C) Touch screen controller dispenses specific inoculum volume in a microinjection syringe into determined region of the mouse brain. (D) Digital display console design ensures full range of motion, angulation, and rotation of the stereotaxic manipulator. (E) Infrared warming pad with touchscreen controller and temperature feedback accurately monitors the mouse’s temperature. (F) Isoflurane anesthesia machine and chamber. (G) LED clamp light lamp with flexible gooseneck. (H) The stainless-steel glass bead bath decontaminates micro-dissecting instruments between procedures. The picture in this figure was taken by Melissa E. Munzen. (TIF) [file pntd.0011068.s001.tif]
